# Blocking Extracellular Chaperones to Improve Cardiac Regeneration

**DOI:** 10.3389/fbioe.2020.00411

**Published:** 2020-05-26

**Authors:** Laura Seclì, Matteo Sorge, Alessandro Morotti, Mara Brancaccio

**Affiliations:** ^1^Department of Molecular Biotechnology and Health Sciences, University of Turin, Turin, Italy; ^2^Department of Clinical and Biological Sciences, University of Turin, Turin, Italy

**Keywords:** extracellular chaperones, HSPs, Toll-like receptors, sterile inflammation, myocardial infarction, cardiomyopathy, heart failure

## Abstract

Chronic or acute insults to the myocardium are responsible for the onset of cardiomyopathy and heart failure. Due to the poor regenerative ability of the human adult heart, the survival of cardiomyocytes is a prerequisite to support heart function. Chaperone proteins, by regulating sarcomeric protein folding, function, and turnover in the challenging environment of the beating heart, play a fundamental role in myocardial physiology. Nevertheless, a number of evidences indicate that, under stress conditions or during cell damage, myocardial cells release chaperone proteins that, from the extracellular milieu, play a detrimental function, by perpetuating inflammation and inducing cardiomyocyte apoptosis. Blocking the activity of extracellular chaperones has been proven to have beneficial effects on heart function in preclinical models of myocardial infarction and cardiomyopathy. The application of this approach in combination with tissue engineering strategies may represent a future innovation in cardiac regenerative medicine.

## Intracellular Chaperones

Sarcomere efficiency is a priority to maintain cardiac function. The impressive sarcomere structure depends on the rigorous association of hundreds of proteins, held together by non-covalent bonds in the challenging context of continuous contractions and relaxations. Indeed, the rhythmic mechanical stretch of the beating heart is, *per se*, a source of protein unfolding and a number of pathological factors, such as pressure overload, metabolic challenges, oxidative stress, may tip the balance toward dysfunction. An efficient quality control mechanism is essential for sarcomere maintenance and dynamic adaptation to new physiological conditions (Willis et al., 2009). Further, unfolded protein degradation through the ubiquitin–proteasome pathway and autophagy (Ranek et al., 2018) is crucial in protecting cardiomyocytes from the accumulation of dangerous protein aggregates. Chaperone proteins are in charge of carrying out these essential tasks and monitoring the efficiency of the entire contractile system. Chaperones have been originally discovered as induced by hyperthermia, whereby the name of heat shock proteins (HSPs) has been attributed to a number of chaperones (Schlesinger, 1990). Later, it became clear that several chaperones are induced upon different types of stress stimuli, including oxidative stress, pathogen infection, mechanical stress, hypoxic conditions, and ischemia (Sbroggio et al., 2008; Tarone and Brancaccio, 2014; Sorge and Brancaccio, 2016; Penna et al., 2018). Chaperones are divided in different subfamilies depending on their structure, molecular weight and on their ability to hydrolyze ATP (Macario and Conway de Macario, 2005; Kampinga et al., 2009). In this review, we will mention chaperones belonging to two subclasses: ATP-dependent chaperones and small heat shock proteins (sHSPs), lacking ATPase activity. The best known ATP-dependent chaperones are the heat shock proteins HSP90, HSP70, and HSP60 (numbers are indicative of their molecular weights). They bind to two classes of proteins: substrate proteins and co-chaperones. Chaperone binding allows substrate proteins, also called clients, to reach their functional conformation, to refold after denaturation and to be protected from degradation. Co-chaperones, instead, assist chaperones in their function, favoring client protein recognition and the transition toward the different structural states required for binding and release of clients. The family of sHSPs is characterized by the absence of ATPase activity, a lower molecular weight, the presence of a highly conserved α-crystallin domain, and their attitude to form oligomers (Carra et al., 2019; Haslbeck et al., 2019). Both ATP-dependent chaperones and sHSPs play a protective role in the myocardium thanks to their ability to cope with protein misfolding, to promote unfolded protein degradation and to support survival signaling in cardiomyocytes (Willis and Patterson, 2013; Tarone and Brancaccio, 2014).

## Extracellular Chaperones

Chaperones are conventionally considered intracellular proteins, prevalently located in the cytoplasm, in the endoplasmic reticulum, in mitochondria, and in the nucleus. In addition to their well-known roles inside the cells, some chaperones and co-chaperones have been found to be secreted in the extracellular milieu in response to cell stress, through an unconventional secretory pathway, and to exert different functions from the outside (Srivastava, 2002b; Calderwood, 2018; Pockley and Henderson, 2018). Among other activities, extracellular chaperones work as damage-associated molecular patterns (DAMPS). As for pathogen-associated molecular patterns (PAMPs), DAMPs are recognized by pattern-recognition receptors (PRRs), such as Toll-like receptors (TLRs), which, in turn, trigger inflammatory responses. In addition to chaperones, DAMPs include the high-mobility group box 1 (HMGB1), S100 family of proteins, RNA, mitochondrial DNA, uric acid, adenosine nucleotides, and others. These molecules may reach the outside by a regulated unconventional secretion induced by stress or may be released during cell damage. From the extracellular milieu, they bind to PRRs on different cell types, activate signaling pathways, and orchestrate cytokine production and immune cell recruitment (Asea et al., 2000; Asea, 2003; Bianchi, 2007). The release of DAMPs plays a role in different diseases in which the immune system is actively involved, including arthritis, multiple sclerosis, cancer, and cardiovascular diseases (Roh and Sohn, 2018). Nevertheless, the release of chaperones from stressed cells may represent an ancient and evolutionary conserved protection mechanism against pathogen infection (Srivastava, 2002a, b). Indeed, chaperones, by binding antigenic peptides and interacting with receptors on antigen-presenting cells, are crucial players in antigen internalization and processing, contributing to innate and adaptive immune responses. Stressful conditions and tissue necrosis, by mediating chaperone release, trigger a sterile inflammation that, besides clearing cellular debris, may extend organ damage and blunt reparative responses (Lai et al., 2019).

The myocardium is composed of different cell types, whereas cardiomyocytes and cardiac fibroblasts are the more represented, endothelial cells, vessel smooth muscle cells, and immune cells are also present in a relevant number. All these cell types express PRRs able to sense DAMPS and detect cardiac stress and damage. After myocardial infarction, the release of DAMPs from injured cardiomyocytes results primarily in the recruitment and activation of innate immune cells, causing endothelial damage and cardiomyocyte injury (Arslan et al., 2011). Accordingly, Toll-like receptor 2 (TLR2) and TLR4 null mice are protected from ischemia–reperfusion injury (Sakata et al., 2007; Arslan et al., 2010, 2011; Li et al., 2011). In addition, DAMPs promote fibroblast proliferation, extracellular matrix deposition, and secretion of metalloproteinases (MMPs) that may jeopardize the stability of the scar (Turner, 2016). Overall, TLR signaling in acute myocardial infarction has been convincingly associated to a maladaptive remodeling and with a decreased cardiac function (Shishido et al., 2003; Satoh et al., 2006b; Timmers et al., 2008; Turner, 2016).

## HSP70

**FIGURE 1:**
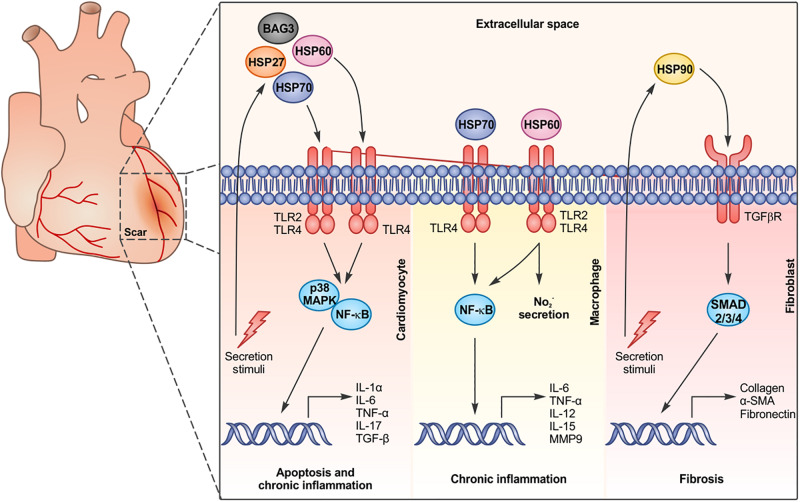
Chaperone proteins are released by cardiac cells and bind to surface receptors on different cell types, activating specific signaling pathways and inducing apoptosis, chronic inflammation, and fibrosis.

HSP70 is an ubiquitously expressed chaperone that, inside the cell, plays an essential role in mediating protein folding and exerts a protective activity against hypoxic and ischemic events (Hecker and McGarvey, 2011). Following different insults such as ischemia, hypoxia, and hemodynamic overload, HSP70 and its cognate protein HSC70 are upregulated in cardiomyocytes and secreted or passively released. Indeed, in mice subjected to abdominal aortic constriction, HSP70 accumulates on the cardiomyocyte plasma membranes and in the mouse serum in a time-dependent manner (Cai et al., 2010). Treatment of mice with doxorubicin, a chemotherapy drug known to cause cardiotoxicity, also induces HSP70 upregulation in the heart and its subsequent accumulation on cardiomyocyte membranes and mouse serum (Liu P. et al., 2019). Moreover, patients experiencing cardiac arrest (Jenei et al., 2013a), acute myocardial infarction (Dybdahl et al., 2005; Satoh et al., 2006a), or coronary artery bypass grafting with the use of cardiopulmonary bypass (Dybdahl et al., 2002) present a higher HSP70 level in the serum, compared to healthy subjects. In these patients, higher circulating levels of HSP70 correlate with increased serum levels of proinflammatory cytokines such as IL-1α, IL-6, TNF-α, IL-17, and TGF-β and myocardial necrosis markers, such as creatine kinase and cardiac troponin T, and are associated with an increased mortality during the follow-up period (Dybdahl et al., 2002, 2005; Satoh et al., 2006a; Jenei et al., 2013a, b). Primary isolated cardiomyocytes treated with recombinant HSP70 proteins show an increase in NF-κB activity, a decreased contractility and a raise in apoptotic death. Stimulation of the cardiac muscle cell line HL-1 with recombinant HSP70 promotes the expression of inflammatory markers downstream the p38 and the NF-κB signaling pathways. The activity of the recombinant HSP70 was totally impaired in TLR2 null cardiomyocytes and in cardiomyocytes deficient for the TLR signal adaptor protein MyD88 (Mathur et al., 2011). Similarly, treatment of mouse cardiomyocytes with recombinant HSC70 induces the phosphorylation of p38; increases the expression of mRNA coding for TNF-α, IL-1β, and IL-6; and depresses cardiac contractility, in a TLR4-dependent manner (Zou et al., 2008; Ao et al., 2009). Overall, these data suggest that once secreted, HSP70 and HSC70 bind to TLR2 and TLR4 expressed on cardiomyocytes and induce the p38 MAPK and the NF-κB signaling pathways (Figure 1). Due to the high expression of TLRs on immune cells, it is not surprising that HSP70 and HSC70 possess a potent immunomodulatory role in the myocardium, regulating immune infiltration after stress stimuli. For instance, binding of a recombinant HSP70 to TLR4 on peritoneal-derived macrophages induces the production of TNF-α (Figure 1), which, in turn, stokes chronic inflammation in the myocardium. In patients with myocardial infarction, HSP70 serum levels are positively correlated with the number of TLR4-positive monocytes in the heart, which favor heart failure progression. The presence of HSP70 in patients’ serum not only represents a reliable marker in heart failure (Jenei et al., 2013b), but may also be exploited as a target to treat cardiac pathologies. The functional antagonism of the extracellular HSP70 (eHSP70), using anti-HSP70 antibodies, significantly attenuates cardiac hypertrophy, fibrosis, and cardiomyocyte apoptosis induced by pressure overload. Moreover, the treatment with HSP70-neutralizing antibodies reduces pro-inflammatory M1 macrophage infiltration in the myocardium, blunting chronic inflammation (Cai et al., 2010; Liu P. et al., 2019). Injection of anti-HSP70 antibodies, after doxorubicin treatment in mice, significantly counteracts α-SMA and collagen-1 upregulation, two important markers of fibrosis in the myocardium, and reduces the production of IL-6, TFG-β, and IL-17A. At the same time, anti-HSP70 antibodies inhibit the expression of inducible NO synthase and cyclooxygenase 2, suggesting that the chronic inflammation in the hearts of doxorubicin-treated mice can be reversed by blocking eHSP70 activity (Liu P. et al., 2019). Similarly, treatment with anti-HSC70 antibodies in mice subjected to ischemia/reperfusion (I/R) improves post-ischemic cardiac functional recovery and reduces the expression of proinflammatory cytokines (IL-6, IL-1β, and TGF-β) (Zou et al., 2008), favoring cardiomyocyte survival. Neutralizing antibodies against TLR2 and TLR4 mimic the effect of HSP70/HSC70 blocking antibodies, preventing reduction in fractional shortening and increasing heart function (Mathur et al., 2011).

## BAG3

Bcl-2-associated athanogene 3 (BAG3) plays crucial roles in cardiomyocytes by inhibiting apoptosis and promoting unfolded protein degradation through macroautophagy. Indeed, mutations in the BAG3 coding gene are associated with dilated cardiomyopathy (Liu L. et al., 2019). Besides this important intracellular role, BAG3 has been found secreted by the rat cardiomyoblast cell line H9c2 and by primary adult human cardiomyocytes in response to stress stimuli and it is detectable in the sera of heart failure patients (De Marco et al., 2013; Gandhi et al., 2015). The extracellular BAG3 triggers the PI3K/AKT/eNOS pathway in endothelial cells, causing nitric oxide release and vasorelaxation (Carrizzo et al., 2016). Of note, BAG3 secreted by cancer cells may activate macrophages and promote IL-6 production, suggesting that BAG3 may regulate inflammation also in the myocardial context (Rosati et al., 2015). Further studies are awaited to define the impact of the extracellular BAG3 on cardiac function after myocardial damage.

## HSP90

HSP90 is an essential and ubiquitous chaperone that exerts multiple roles inside the cells, ranging from protein folding, buffering protein denaturation, and assisting signal transduction protein conformational changes (Schopf et al., 2017). HSP90 is secreted in response to different stress stimuli and, from the extracellular milieu, it is able to bind to several cellular receptors, among them TLR2 and 4, and to unleash intracellular signal transduction pathways (Calderwood et al., 2007). The role of the extracellular HSP90 (eHSP90) has been extensively studied in the cancer microenvironment, where it regulates metalloproteinase activity (Baker-Williams et al., 2019) and fibronectin matrix assembly (Chakraborty et al., 2020) and promotes cancer cell survival, migration, and invasion (Calderwood, 2018). In this context, eHSP90 can also recruit and activate stromal fibroblasts (Bohonowych et al., 2014; Tang et al., 2019). Garcia and colleagues described cardiac fibroblasts as a primary source of eHSP90 in the myocardium subjected to pressure overload. Cardiac fibroblasts are activated by myocardial damage or stress and are responsible for matrix deposition. eHSP90 has been found to interact with the transforming growth factor-β receptor I (TGFβRI) on the surface of cardiac fibroblasts, inducing TGF-β signaling and promoting collagen production and fibrosis. Indeed, mice null for the inducible HSP90α isoform show reduced collagen deposition and cardiac fibrosis in long-term pressure overload (Garcia et al., 2016; Figure 1).

## HSP60

HSP60 is a chaperone protein located both in mitochondria and in the cytosol, responsible for the folding of mitochondrial-imported proteins (Singh et al., 1990; Brinker et al., 2001). In mitochondria, HSP60 monomers assemble in a double heptameric ring, associated with a co-chaperone, HSP10, that caps the inner cavity and regulates substrate processing (Xu et al., 1997). Instead, in the cytosol, HSP60 is present as a monomer or bound to specific interactors, as the pro-apoptotic protein Bax (Kirchhoff et al., 2002). Several studies demonstrated that the expression and distribution of HSP60 is altered in cardiac diseases (Novo et al., 2011). In a rat model of heart failure, HSP60 was found upregulated after ligation of the left anterior descending artery, together with proinflammatory cytokines, brain, and atrial natriuretic peptides (Lin et al., 2007). In isolated cardiac myocytes subjected to ischemic insults, HSP60 translocates from the cytosol to plasma membrane lipid rafts and is secreted and released in exosomes (Gupta and Knowlton, 2002; Lin et al., 2007). HSP60 doubles in patients affected by ischemic disease, if compared with healthy controls (Knowlton et al., 1998), and its membrane translocation and release have been also demonstrated (Lin et al., 2007; Tian et al., 2013). Moreover, HSP60 circulating levels in patients with coronary artery disease and acute myocardial infarction correlate with the extent of the disease (Mandal et al., 2006; Novo et al., 2011).

The translocation of HSP60 to the plasma membrane and its secretion induce the release of Bax that can move to mitochondria and activate the apoptotic cascade (Gupta and Knowlton, 2002). In addition, HSP60 actively triggers apoptosis in cardiomyocytes from the extracellular compartment (Kim et al., 2009; Li et al., 2011). Indeed, in adult-rat cardiomyocytes or H9c2 cells, the extracellular HSP60 (eHSP60) binds to TLR4, but not TLR2, and promotes the expression of TNF-α, IL-6, and IL-1β (Kim et al., 2009; Tian et al., 2013; Figure 1). Pretreatment of cells with blocking antibodies against TLR4 or inhibitors for MyD88 significantly decreases cardiomyocyte apoptosis, while antibodies against TLR2 have no effect (Kim et al., 2009). Antibodies against TNF-α, but not against IL-1β, also block eHSP60-induced apoptosis (Kim et al., 2009). All these evidence suggest that extracellular eHSP60 specifically activates the TLR4-MyD88-NF-κB pathway, thus inducing TNF-α-mediated cardiomyocyte apoptosis. *In vivo* coronary artery ligation followed by reperfusion induces the activation of the interleukin receptor-associated kinase-1 (IRAK-1), a kinase critical for TLR signaling. The treatment of mice with anti-HSP60 antibodies, prior to the ligation, significantly attenuates IRAK-1 activation (Li et al., 2011). The absence of TLR4 or MyD88, but not TLR2, impairs IRAK-1 activation in response to I/R, confirming the specificity of eHSP60 in activating TLR4 signaling (Li et al., 2011). eHSP60 induces caspase-8-dependent apoptosis and the absence of TLR4, or the treatment of mice with anti-HSP60 antibodies, and attenuates I/R-induced cell death (Li et al., 2011).

In endothelial cells, HSP60 elicits the expression of E-selectin, ICAM-1, and VCAM-1, favoring the leukocyte trafficking within the vascular wall (Kol et al., 1999). In macrophages, it has been suggested that HSP60 binds to TLR2 and TLR4 and triggers an intracellular signal via MyD88 and TRAF6 (Vabulas et al., 2001), leading to the release of NO_2_^–^, the induction of TNF-α and IL-6 and the overexpression of IL-12 and IL-15 (Chen et al., 1999; Kol et al., 1999; Figure 1). HSP60 has also been found to localize in the atherosclerotic plaques, where it can provoke the production of TNF-α and MMP9 by macrophages (Kol et al., 1998). In addition, HSP60 can act as an autoantigen during chronic inflammation, as suggested by the presence of antibodies and T-cell responses to HSP60 in various inflammatory conditions (Nomoto and Yoshikai, 1991; Res et al., 1991), causing cardiac decline (Ohashi et al., 2000; Burian et al., 2001; Wysocki et al., 2002).

## HSP27

HSP27 is a widely expressed chaperone protein belonging to the small HSP family, exerting a number of protective function in cardiomyocytes (Tarone and Brancaccio, 2014). Human and murine hearts release HSP27 in the circulation after myocardial infarction and I/R. Treatment of isolated mouse hearts with HSP27 recombinant proteins induces NF-κB activation and IL-6 production in the myocardium and causes a depression in cardiac function. Treatment with recombinant HSP27 activates an inflammatory response also in human and murine coronary vascular endothelial cells, promoting the overexpression of ICAM-1, MCP-1, IL-6, and IL-8 in a dose-dependent manner. All these effects are mediated by TLR2 and 4, since the treatment with HSP27 has no effect on cells derived from TLR2-null or TLR4-defective mice (Figure 1). Of note, neutralizing antibodies against HSP27 reduce myocardial NF-κB activity and IL-6 production and improve functional recovery after cardiac I/R (Jin et al., 2014).

## Conclusion

**FIGURE 2:**
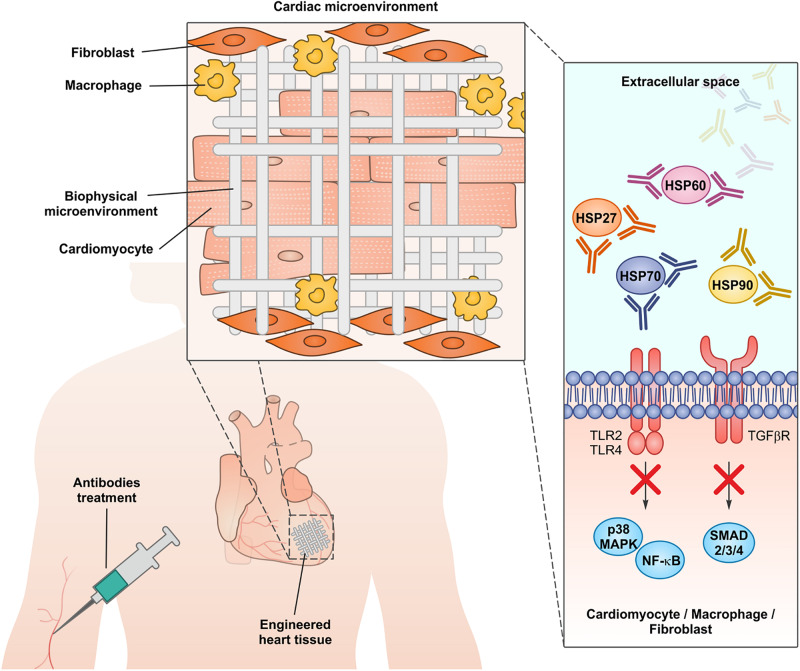
The administration of neutralizing antibodies against extracellular chaperones, in conjunction with the implantation of engineered cardiac tissues, may represent a new approach to dampen myocardial inflammation and improve the engraftment.

All the evidence discussed above indicate that chaperones are actively secreted from stressed cells or released from damaged cells during chronic and acute cardiac insults or during clinical procedures like coronary artery bypass graft (Westaby, 1987; Levy and Kelly, 1993; Szerafin et al., 2008; Khan et al., 2014). Of note, the global inhibition of chaperone functions in heart using small molecules able to cross the plasma membrane would be seriously harmful, since chaperones are crucial in maintaining cardiomyocyte proteostasis and in sustaining heart function both in healthy conditions and during pathological insults (Willis et al., 2009; Willis and Patterson, 2013; Tarone and Brancaccio, 2014; Sorge and Brancaccio, 2016; Penna et al., 2018). A number of therapeutic antibodies have already been approved for clinical applications and many are in late-stage trials mainly to treat cancer and autoimmune diseases. Antibodies may block the activity of extracellular proteins, without affecting the intracellular counterparts (Redman et al., 2015). Consistently, antibodies able to blunt the detrimental activity of extracellular chaperones have been used successfully in preclinical models of myocardial infarction and cardiomyopathy (Zou et al., 2008; Cai et al., 2010; Li et al., 2011; Jin et al., 2014; Liu P. et al., 2019). Research in developing engineered heart tissues to improve myocardial regeneration is an active field in regenerative medicine. The identification of suitable biocompatible materials to be colonized with functional cardiomyocytes is a promising strategy to heal an organ with a very poor regenerative potential as the human heart (Madonna et al., 2019). Nevertheless, human cardiomyocytes subjected to stress locally activate innate and adaptive immunity through chaperone release (Levy and Kelly, 1993; Veres et al., 2002; Wysocki et al., 2002; Mandal et al., 2006; Szerafin et al., 2008; Novo et al., 2011). This chronic sterile inflammation in the damaged myocardium may represent an issue in engineered tissue engraftment, by promoting apoptosis in colonizing cardiomyocytes and increasing the probability of rejection. We propose that the inhibition of extracellular chaperones during the implant of engineered heart tissues may represent a new advance in improving grafting and heart regeneration (Figure 2).

## Author Contributions

All authors wrote the manuscript and approved the contents for publication.

## Conflict of Interest

The authors declare that the research was conducted in the absence of any commercial or financial relationships that could be construed as a potential conflict of interest.
